# Supplementary material for: Beyond recombination: Exploring the impact of meiotic frequency on genome-wide genetic diversity
Source: PLoS Genet. 2025 Aug 4;21(8):e1011798. doi: 10.1371/journal.pgen.1011798 (PMC12408092; doi:10.1371/journal.pgen.1011798)
Supplement: S1 File — Times to fixation of the beneficial mutation for different α values. Fig B. The effects of the two models of α with hard selective sweeps on genetic diversity (π). Fig C. Times to fixation of the beneficial mutations under different strengths of selection. Fig D. The effect of the recombination rate per basepair on π/π0, with s = 0.05 and h = 0.5. Fig E. Effect of ρ versus ρα on π/π0. Fig F. Effects of the dominance coefficient h. Fig G. The behavior of π/π0 with different dominance coefficients (h) and frequencies of meiosis between theoretical model and simulation. Fig H. Times to fixation of beneficial mutations under different recombination rates. Fig I. Effect of ρ versus ρα on the time to fixation of a beneficial mutation. Fig J. Times to fixation of a beneficial mutation with different dominance coefficients. Fig K. Effects of the selfing rate. (PDF) [file pgen.1011798.s004.pdf]

S1 Supplementary Figures

S1 Fig. Times to fixation of the beneficial mutation for different *α* values

S2 Fig. The effects of the two models of *α* with hard selective sweeps on genetic diversity (*π*)

S3 Fig. Times to fixation of the beneficial mutations under different strengths of selection.

S4 Fig. The effect of the recombination rate per basepair on π/π_0, with *s* = 0.05 and *h* = 0.5.

S5 Fig. Effect of *ρ* versus ρ_α on π/π_0.

S6 Fig. Effects of the dominance coefficient *h*.

S7 Fig. The behavior of π/π_0 with different dominance coefficients (*h*) and frequencies of meiosis between theoretical model and simulation

S8 Fig. Times to fixation of beneficial mutations under different recombination rates.

S9 Fig. Effect of *ρ* versus ρ_α on the time to fixation of a beneficial mutation.

S10 Fig. Times to fixation of a beneficial mutation with different dominance coefficients.

S11 Fig. Effects of the selfing rate.

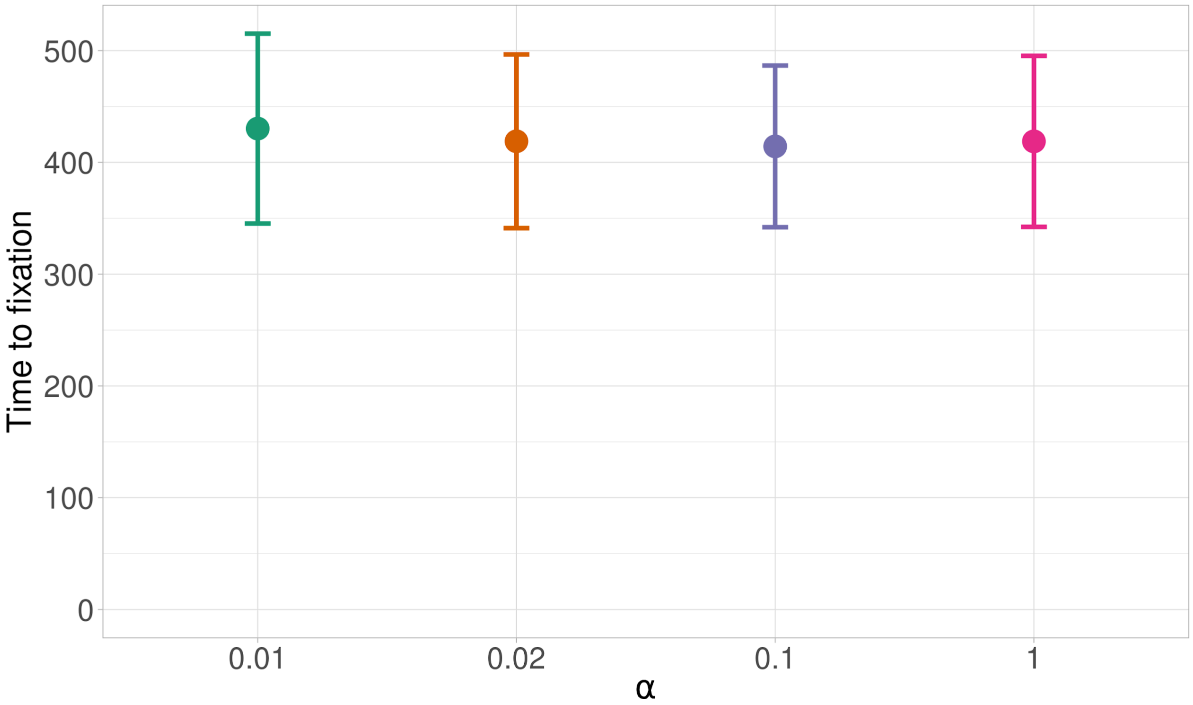

S1 Fig. Times to fixation of the beneficial mutation for different *α* values.

Each case used the following settings: a beneficial mutation was introduced into one individual and the population was allowed to evolve until its fixation, with $\rho = 5\times10^{-8}$, s = 0.05 and h = 0.5. If the beneficial mutation was lost, the simulation was rerun. Dots represent the average time to fixation from 500 simulations, with α = 0.01 (green), α = 0.02 (orange), α = 0.1 (blue), and α = 1 (pink). The error bars are standard errors (see Material and Methods for details).

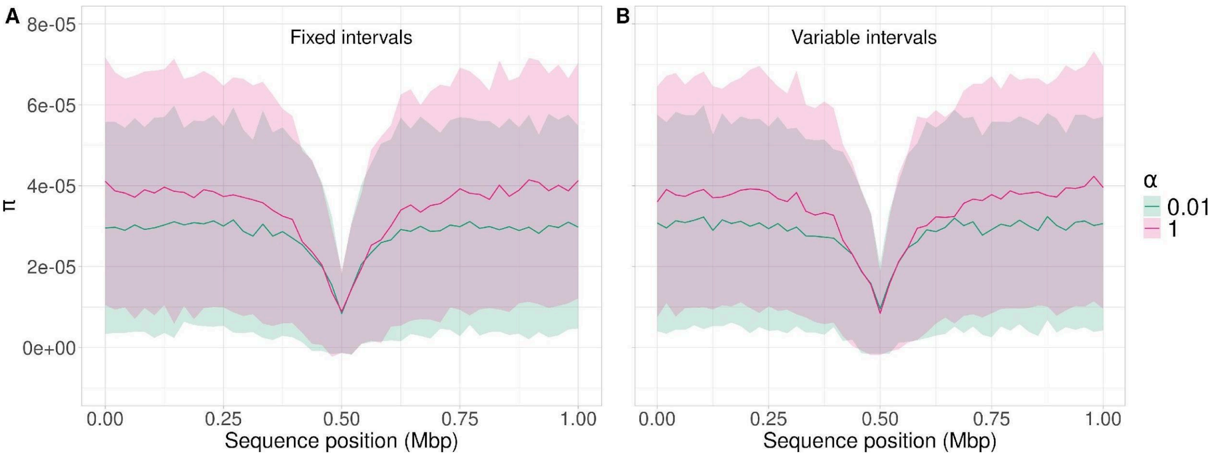

S2 Fig. The effects of the two models of *α* with hard selective sweeps on genetic diversity (*π*) along a 1 Mb chromosome.

The same procedure for introducing a beneficial mutation as in S1 Fig was used, with h = 0.5 and s = 0.05. Colors and lines are the same as in Fig 1. With α = 0.01, the interval between two sexual generations was fixed at 1/α = 100 generations (A) or sampled from a Gaussian distribution centered around 1/α = 100 and a standard deviation of 10 (B)

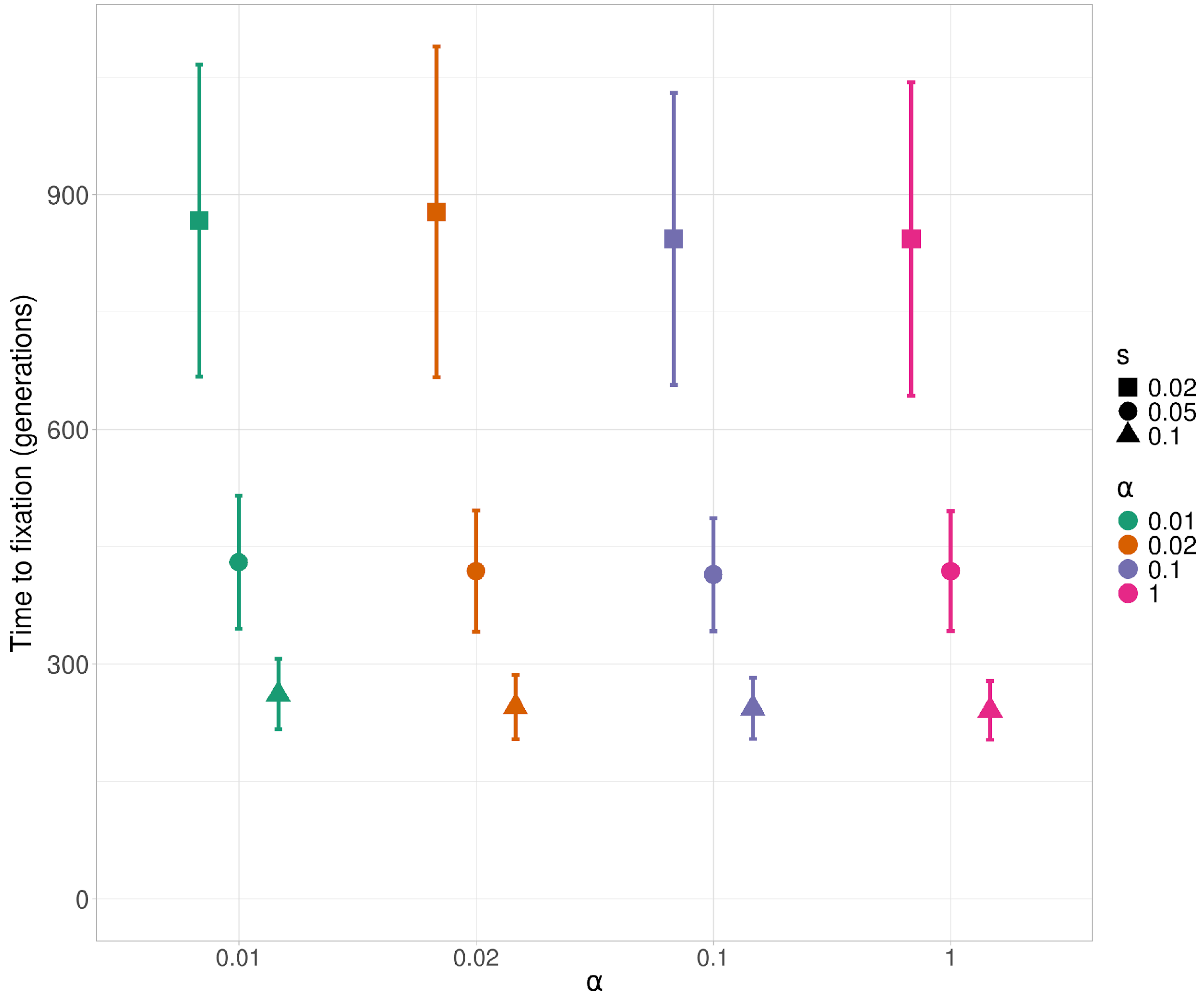

S3 Fig. Times to fixation of the beneficial mutations under different strengths of selection.

The same procedure for introducing a beneficial mutation as in the other figures was used, with ρ = 5 × 10^{-8}, h = 0.5 and a given s. Dots represent the average time to fixation from 500 simulations with α = 0.01 (green), α = 0.02 (orange), α = 0.1 (blue), and α = 1 (pink) and error bars are standard errors (see Material and Methods for details).

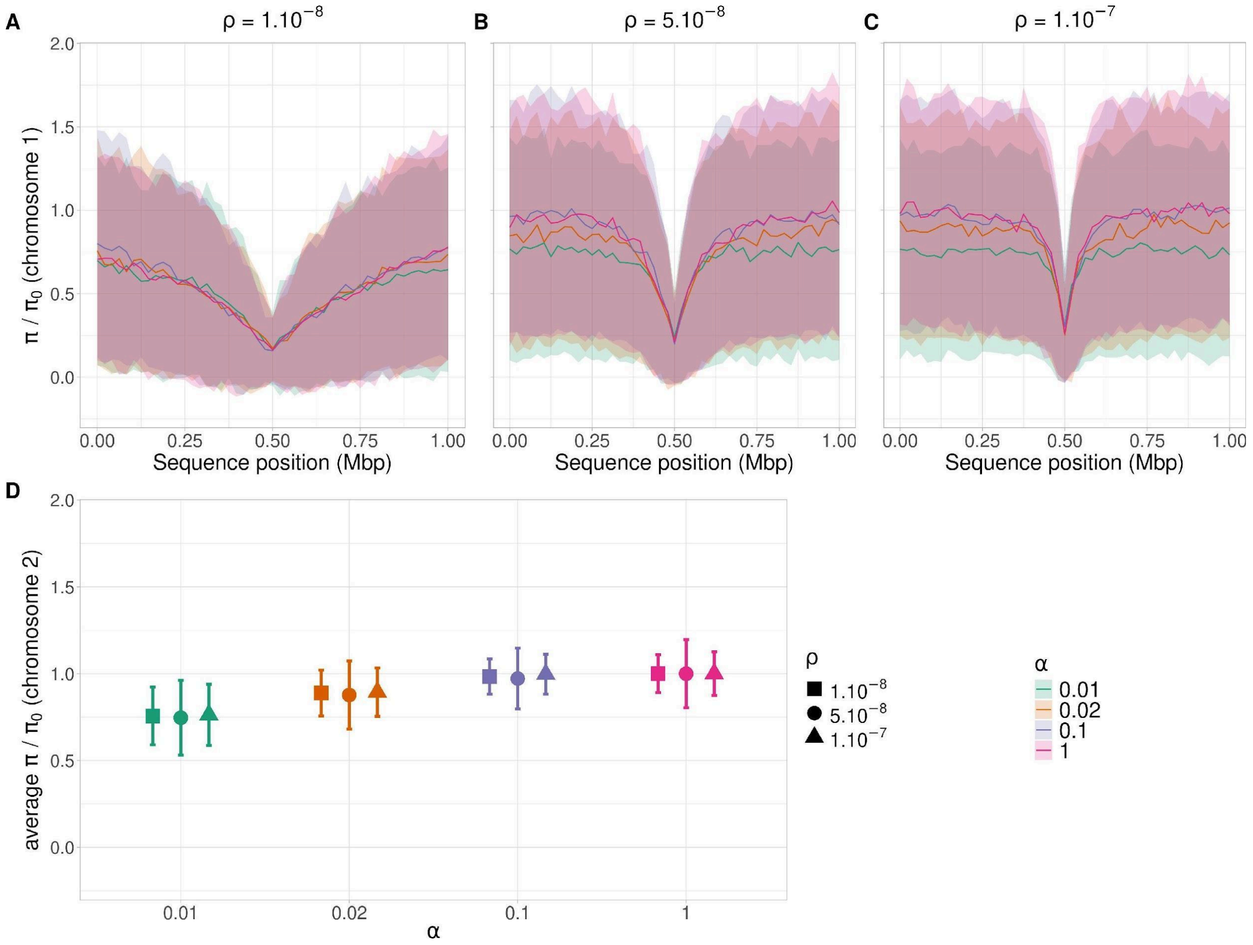

S4 Fig. The effect of the recombination rate per basepair on π/π_0, with s = 0.05 and h = 0.5.

(A-C) π/π_0 along a 1Mb chromosome with a hard sweep at 0.5 Mbp. (D) Average π/π_0 and its standard error for a second chromosome that is unlinked to the first one and which carries only neutral mutations. For $\alpha < 1$, the interval between two sexual generations was drawn from a normal distribution centered on $1/\alpha$ generations (see Material and Methods for details). The same procedure for introducing a beneficial mutation as in the other figures was used. The three cases have different recombination rates per generation: $\rho = 1 \times 10^{-8}$ (A), $\rho = 5 \times 10^{-8}$ (B), and $\rho = 1 \times 10^{-7}$ (C). Colored lines are the same as in Fig 2.

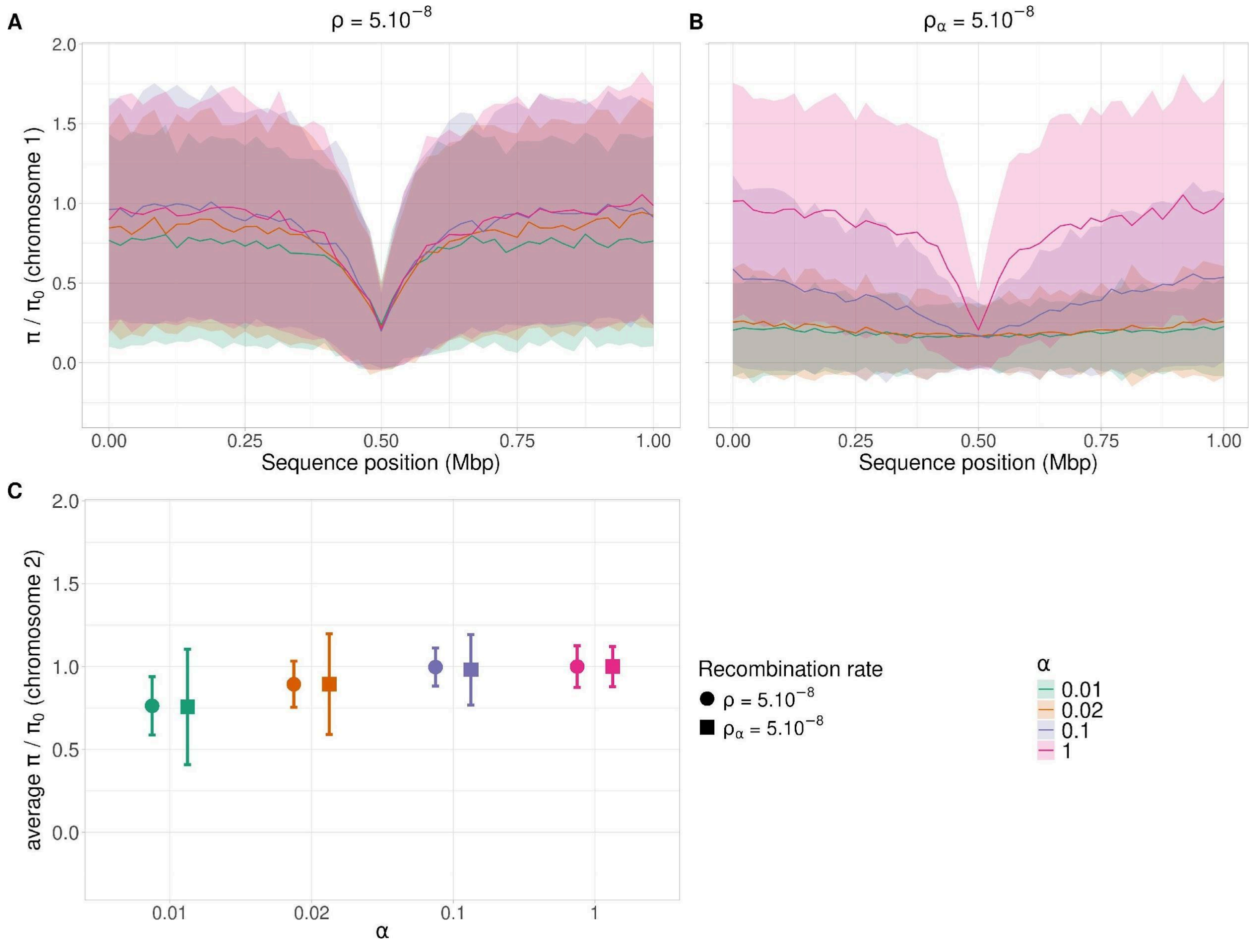

S5 Fig. Effect of ρ versus ρ_α on π/π_0.

(A-B): π/π_0 along a 1Mb chromosome with a selective sweep at 0.5 Mbp, with h = 0.5, s = 0.05. (A) Recombination rate per site per generation $\rho = \alpha\,\rho_\alpha$ is constant at 5×10^{-8} whereas $\rho_\alpha = 5 \times 10^{-6}$ for $\alpha = 0.01$, $\rho_\alpha = 2.5 \times 10^{-6}$ for $\alpha = 0.02$, $\rho_\alpha = 5 \times 10^{-7}$ for $\alpha = 0.1$ and $\rho_\alpha = 5 \times 10^{-8}$ for $\alpha = 1$. (B) Recombination rate per site per meiosis ρ_α is constant at 5×10^{-8} whereas $\rho = 5 \times 10^{-10}$ for $\alpha = 0.01$, $\rho = 1 \times 10^{-9}$ for $\alpha = 0.02$, $\rho = 5 \times 10^{-9}$ for $\alpha = 0.1$ and $\rho = 5 \times 10^{-8}$ for $\alpha = 1$. (C) Average π/π_0 and its standard error for a second, independent chromosome carrying only neutral mutations when ρ is held fixed at the same value (circles) or ρ_α is held fixed (squares). For $\alpha < 1$, the interval between two sexual generations was drawn from a normal distribution centered on $1/\alpha$ generations (see Material and Methods for details). Colored lines are the same as in Fig 2. The same procedure for introducing a beneficial mutation as in other figures was used.

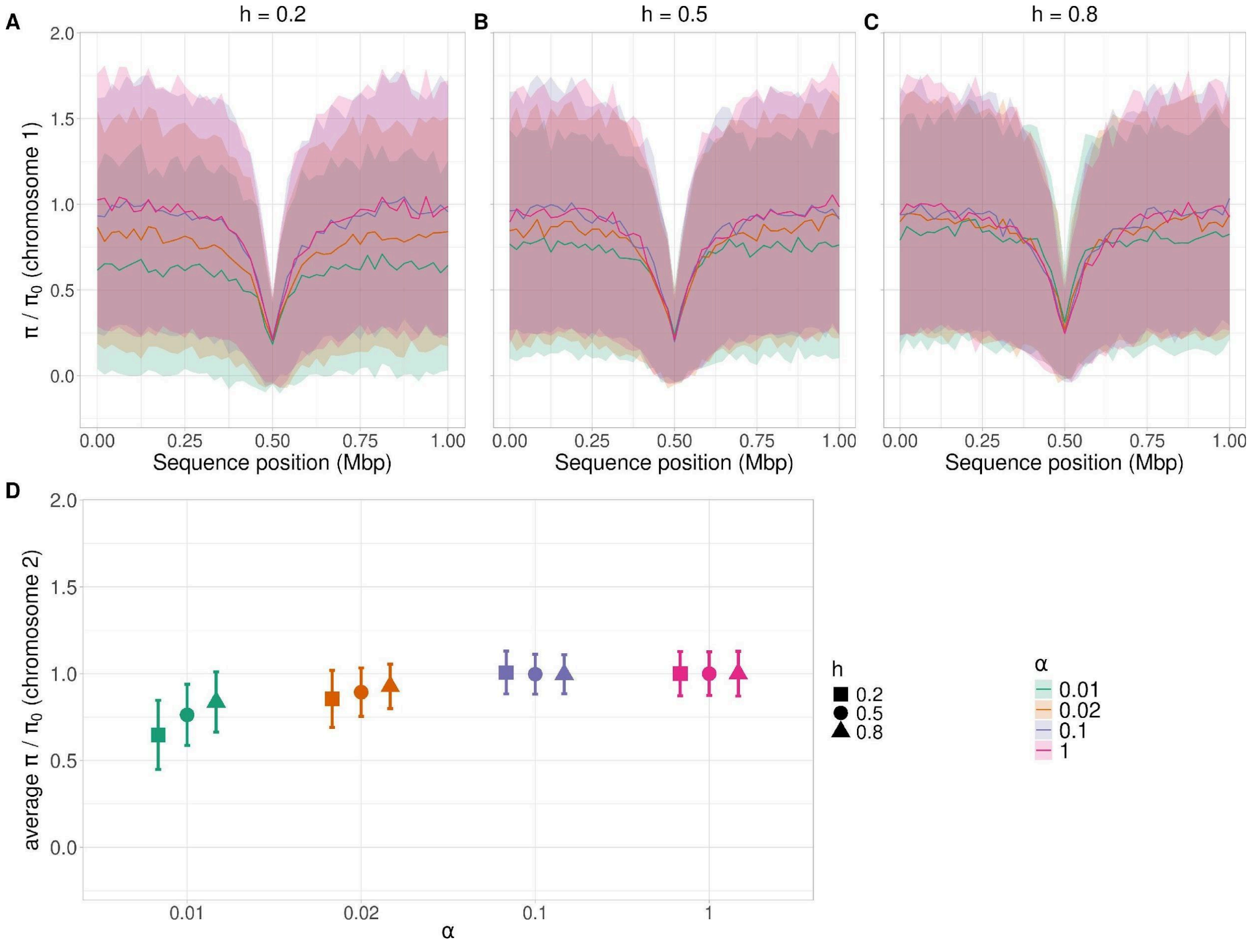

S6 Fig. Effects of the dominance coefficient *h*.

(A)-(C) Values of π/π_0 along a 1Mb chromosome that carries a beneficial mutation (s = 0.05) at 0.5 Mbp with h = 0.2 (panel A), 0.5 (B), and 0.8 (C) and $\rho = 5 \times 10^{-8}$. (D) The mean value of π/π_0 (with standard error) on a second, independent chromosome. The same procedure for introducing a beneficial mutation as in other figures was used. For α < 1, the interval between two sexual generations was drawn from a normal distribution centered on $1/\alpha$ generations (see Material and Methods for details). Colors and lines are the same as in Fig 1.

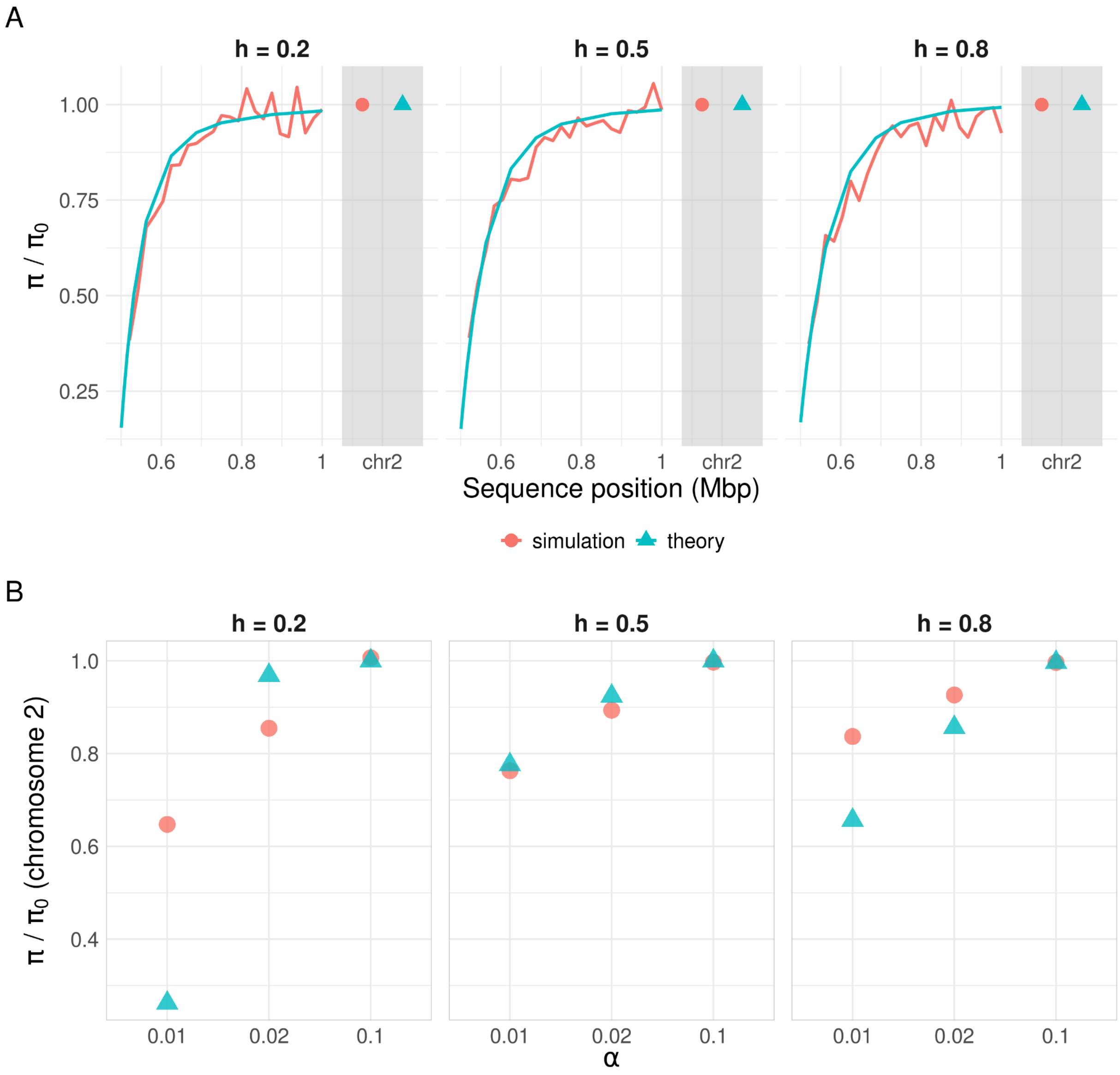

S7 Fig. The behavior of π/π_0 with different dominance coefficients (*h*) and frequencies of meiosis between theoretical model (Equations 14 from [29], in blue and simulation results [from Fig 2] in red).

π/π_0 was computed in the same way as in Fig 2 (see Methods). (A) Comparisons for chromosome 1 (linked) and 2 (unlinked) for $\alpha = 1$, $s = 0.05$. (B) Comparisons for chromosome 2, with a combination of α and h. Values for simulated data (red) are the averages from 500 simulations. The same procedure for introducing a beneficial mutation as in other figures was used.

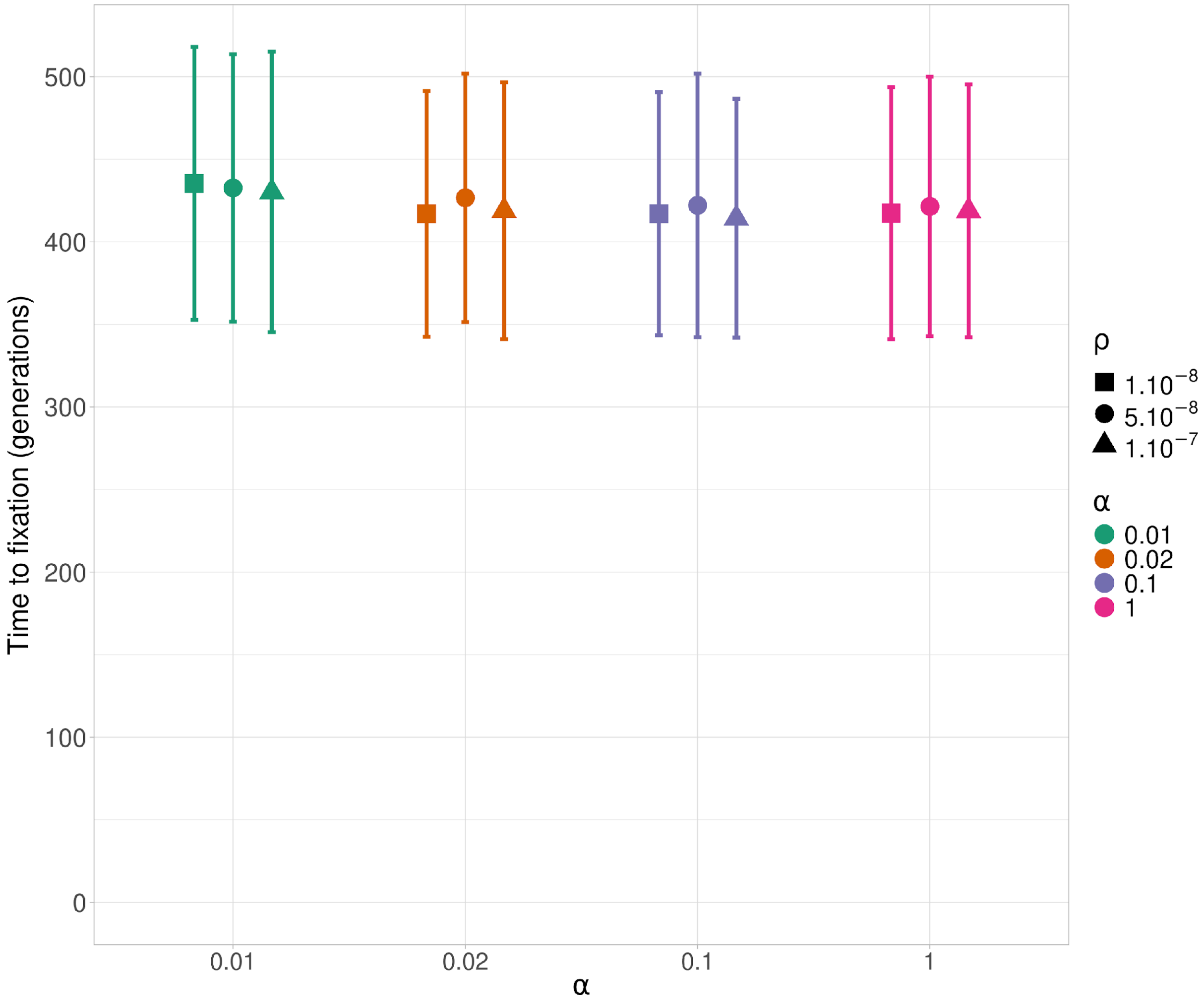

S8 Fig. Times to fixation of beneficial mutations under different recombination rates. The same procedure for introducing a beneficial mutation as in other figures was used, with h = 0.5, s = 0.05 and a given value of ρ. Dots represent the average time to fixation from 500 simulations time with α = 0.01 (green), α = 0.02 (orange), α = 0.1 (blue), and α = 1 (pink), and error bars are standard deviation (see. Material and Methods for details).

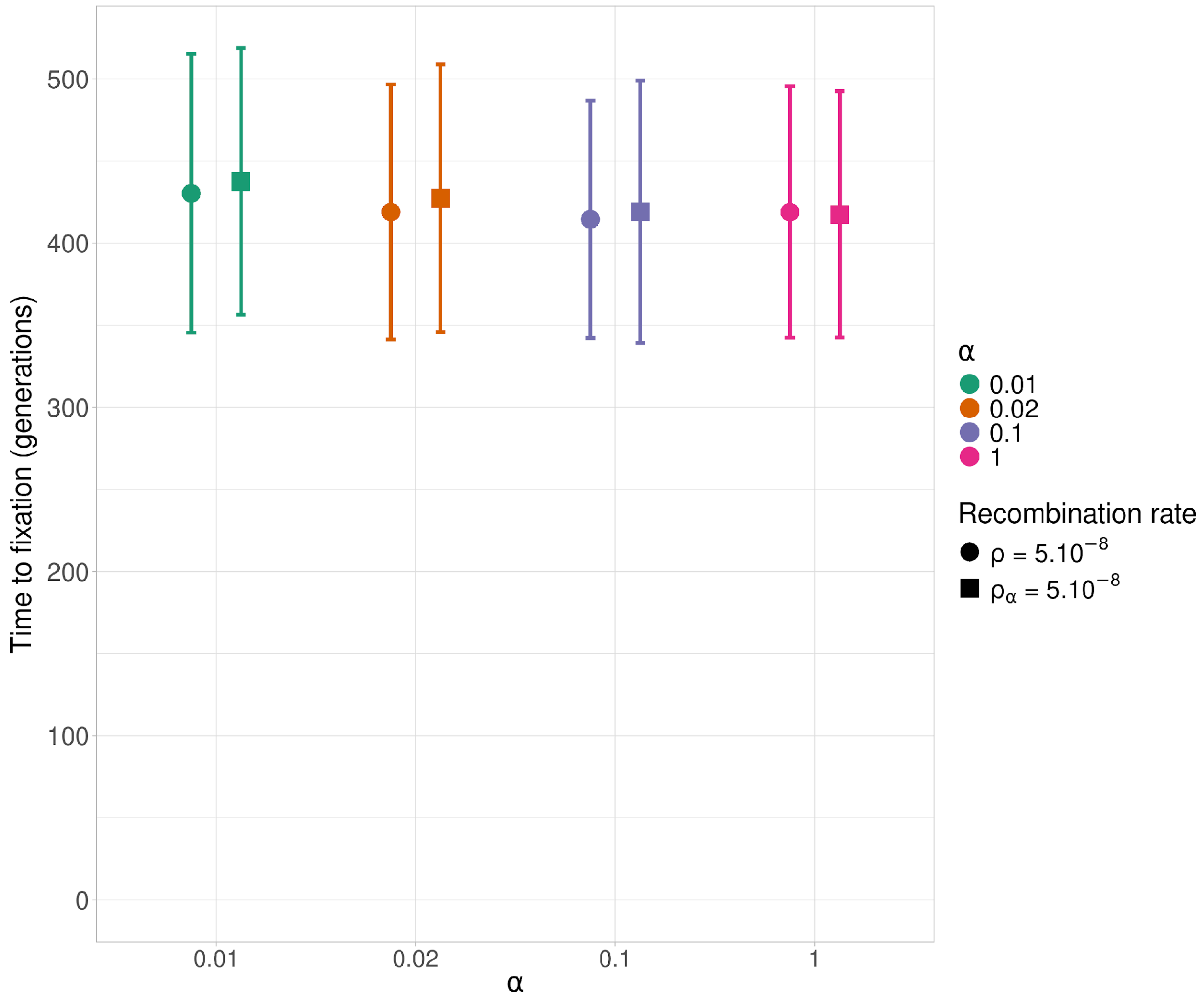

S9 Fig. Effect of ρ versus ρ_α on the time to fixation of a beneficial mutation.

The same procedure for introducing a beneficial mutation as in other figures was used, with h = 0.5, s = 0.05. Either ρ was fixed (circles) or ρ_α was fixed (squares). If recombination rate per site per generation $\rho = \alpha \rho_\alpha$ is fixed at 5×10^{-8}, we have $\rho_\alpha = 5 \times 10^{-6}$ for $\alpha = 0.01$, $\rho_\alpha = 2.5 \times 10^{-6}$ for $\alpha = 0.02$, $\rho_\alpha = 5 \times 10^{-7}$ for $\alpha = 0.1$ and $\rho_\alpha = 5 \times 10^{-8}$ for $\alpha = 1$. Dots represent the average time to fixation from 500 simulations time with $\alpha = 0.01$ (green), $\alpha = 0.02$ (orange), $\alpha = 0.1$ (blue), and $\alpha = 1$ (pink), and error bars are standard errors (see. Material and Methods for details).

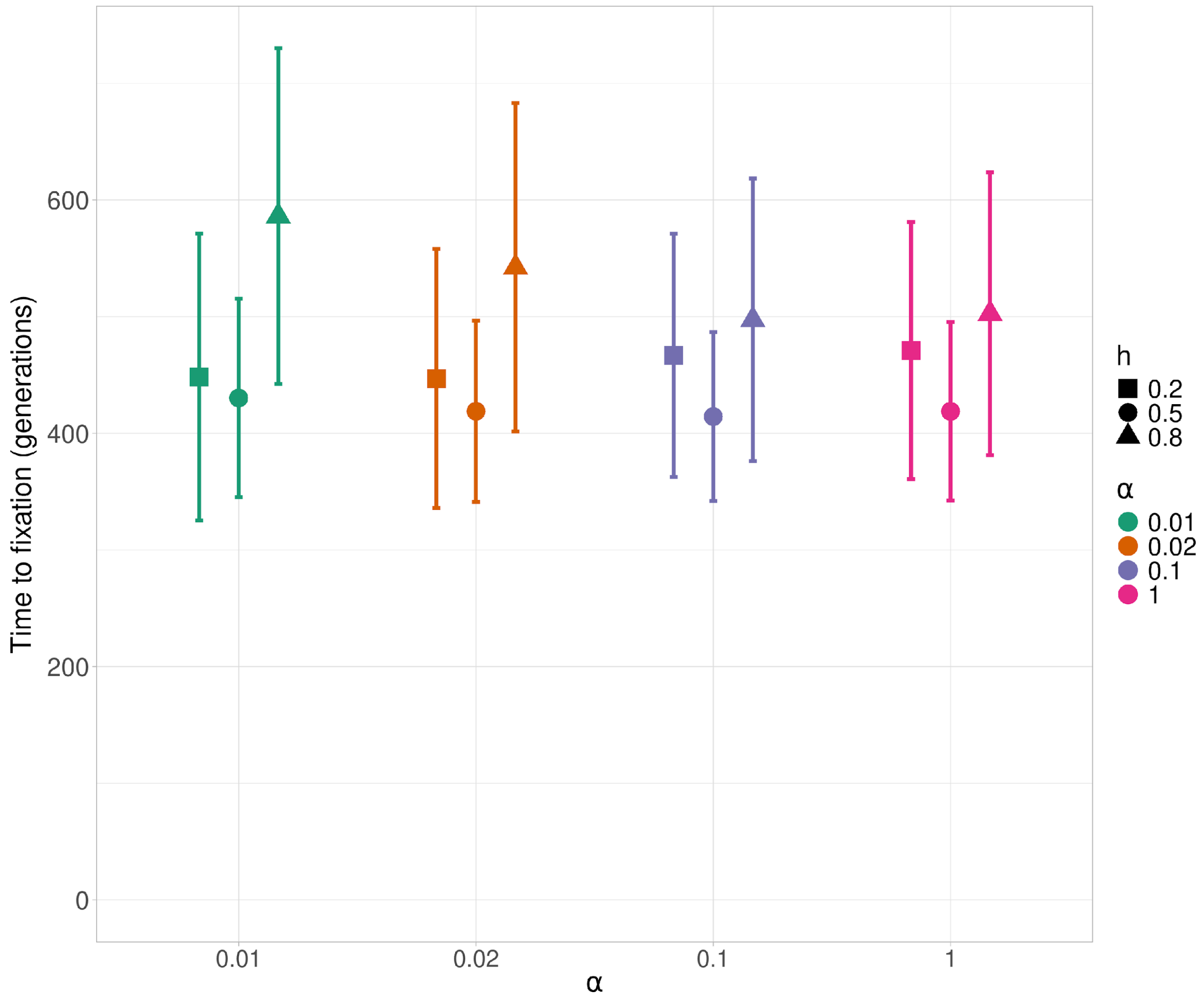

S10 Fig. Times to fixation of a beneficial mutation with different dominance coefficients.

The same procedure for introducing a beneficial mutation as in other figures was used, with $\rho = 5 \times 10^{-8}$, $s = 0.05$ and a given h. Dots represent the average time to fixation from 500 simulations time with $\alpha = 0.01$ (green), $\alpha = 0.02$ (orange), $\alpha = 0.1$ (blue), and $\alpha = 1$ (pink); error bars are standard errors (see. Material and Methods for details).

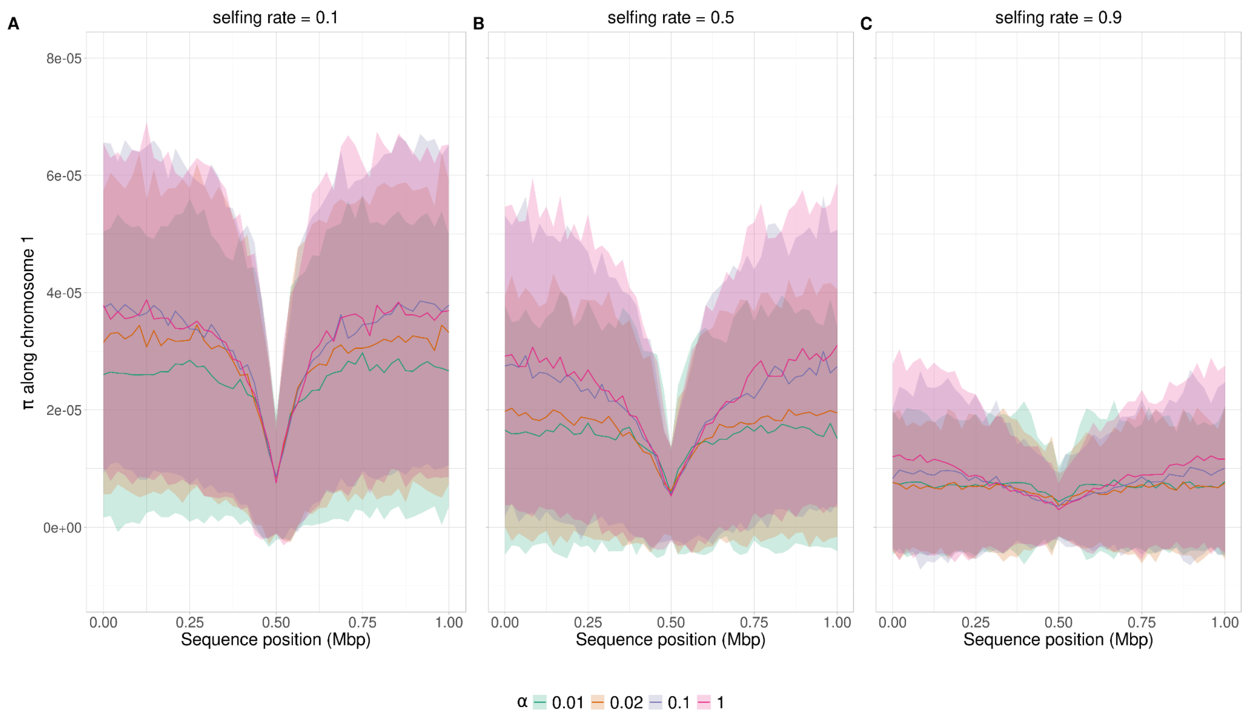

S11 Fig. Effects of the selfing rate.

(A)-(C) Values of π along a 1Mb chromosome that carries a beneficial mutation (s = 0.05, h = 0.5) at 0.5 Mbp with selfing rate = 0.1 (panel A), 0.5 (B), and 0.9 (C) and $\rho = 5 \times 10^{-8}$. The same procedure for introducing a beneficial mutation as in other figures was used. For $\alpha < 1$, the interval between two sexual generations was drawn from a normal distribution centered on $1/\alpha$ generations (see Material and Methods for details). Colors and lines are the same as in Fig 1.
